# Ileal antimicrobial peptide expression is dysregulated in old age

**DOI:** 10.1186/s12979-017-0101-8

**Published:** 2017-08-29

**Authors:** Sarah Tremblay, Nathalie Marie Louise Côté, Guillaume Grenier, Gabriella Duclos-Lasnier, Louis-Charles Fortier, Subburaj Ilangumaran, Alfredo Menendez

**Affiliations:** 1 Department of Microbiology and Infectious Diseases, Faculty of Medicine and Health Sciences, Université de Sherbrooke, 3201 rue Jean-Mignault, Sherbrooke, Québec, J1E 4K8 Canada; 2 Department of Obstetrics and Gynecology, Faculty of Medicine and Health Sciences, Université de Sherbrooke, 3201 rue Jean-Mignault, Sherbrooke, Québec, J1E 4K8 Canada; 3 Department of Surgery, Faculty of Medicine and Health Sciences, Université de Sherbrooke, 3201 rue Jean-Mignault, Sherbrooke, Québec, J1E 4K8 Canada; 4 Department of Pediatrics, Immunology Division, Faculty of Medicine and Health Sciences, Université de Sherbrooke, 3201 rue Jean-Mignault, Sherbrooke, Québec, J1E 4K8 Canada

**Keywords:** Intestinal antimicrobial peptides, Aging, Microbiota, Ileum, Paneth cells, Goblet cells

## Abstract

In an effort to understand the mechanisms underlying the high prevalence of gastrointestinal tract disorders in old age, we investigated the expression of intestinal antimicrobial peptides in the terminal small intestine of aged mice. Our results show that old mice have reduced transcript levels of ileal α-defensins and lysozyme, two important types of intestinal antimicrobial peptides produced by Paneth cells. In contrast, expression of the C-type lectins Reg3b and Reg3g, as well as β-defensin 1, angiogenin 4 and Relmb, which are made by several epithelial cell types, was significantly upregulated in aged animals suggesting an ongoing response to epithelial distress. Those changes in antimicrobial peptide gene expression associated with histological damage of the ileal epithelium and subtle modifications in the composition of the commensal microbiota. Our findings suggest that dysregulation of antimicrobial peptides expression is a feature of homeostasis disruption in the aged intestine and may contribute to geriatric gastrointestinal dysfunction.

Aging is associated with a higher frequency of disorders of the gastrointestinal tract (GIT), which are important causes of morbidity in the elderly population [1]. The GIT is constantly exposed to dietary antigens and trillions of commensals and pathogenic microorganisms, which pose a tremendous immunological challenge. The intestinal epithelium deals with this challenge via the intestinal epithelial barrier, a functional entity composed by the epithelial cells, the mucus layer, the mucosal lymphoid tissue, a full repertoire of effector immune cells, and secreted immunoglobulins and antimicrobial peptides and proteins (AMPP) [2]. Defects of the intestinal epithelial barrier integrity may lead to increased permeability and inflammation [2, 3] and have been proposed as important contributing factors to geriatric gastrointestinal dysfunction [4, 5].

It is not currently known whether alteration in the synthesis of intestinal AMPP is a distinctive feature of gastrointestinal aging. Intestinal AMPP are produced by epithelial cells of the GIT [6]. AMPP have been associated with the control of commensal microbes [7, 8] as well as the defense from enteric infections [9–11], they can affect the composition of the intestinal microbiota and thus, its many functions in host’s metabolism and physiology [12]. AMPP are critical for the maintenance of the intestinal barrier and the immunological homeostasis of the GIT.

We used aged and young C57BL/6 mice (104 and 20 weeks average age, respectively) as a model to investigate changes in the baseline synthesis of ileal AMPP in old age. The groups were composed each of eight females and eight males, for a total of sixteen animals per age group. All animals used in this work were naive and apparently healthy at the time of the study. Ilea from aged mice showed distinct histological features, characterized by a reduction in the number and length of villi (Fig. 1a-c), various degrees of epithelial villi degeneration, generally more pronounced in females, (Fig. 1d-g) and ileal crypt deepening and ballooning (Fig. 1f, g). Atypical goblet-like cells containing Paneth cell-like eosinophilic granules were observed close to the tip of the villi (Fig. 1h), likely representing cells of the secretory lineage that migrated towards the villi tip but failed to undergo terminal differentiation. The secretory granules of Paneth cells appeared larger and very prominent in aged mice (Fig. 1f, j-l) and were surrounded by a thick layer of (unidentified) dense material, distinguishable at the ultrastructural level (Fig. 1j-l). Old mice showed a slight increase in the number of Paneth cells per crypt (Fig. 1m) and goblet cells per villus (Fig. 1n-q). Alcian blue staining revealed larger goblet cell mucin granules, also more intensely stained (Fig. 1o-q) indicating an increase in mucin abundance in the ilea of old mice. The differences in mucin content were not due to differential *Muc2* gene transcription, as equal transcript levels of *Muc2* were found by qPCR in young and old animals (data not shown) indicating regulation at the post-transcriptional level, possibly defective secretion leading to intracellular accumulation of mucins. Such a disparity between *Muc2* transcription and mucin levels/mucus distribution has been observed by others in Reg3g-defficient mice [13].

**Fig. 1:**
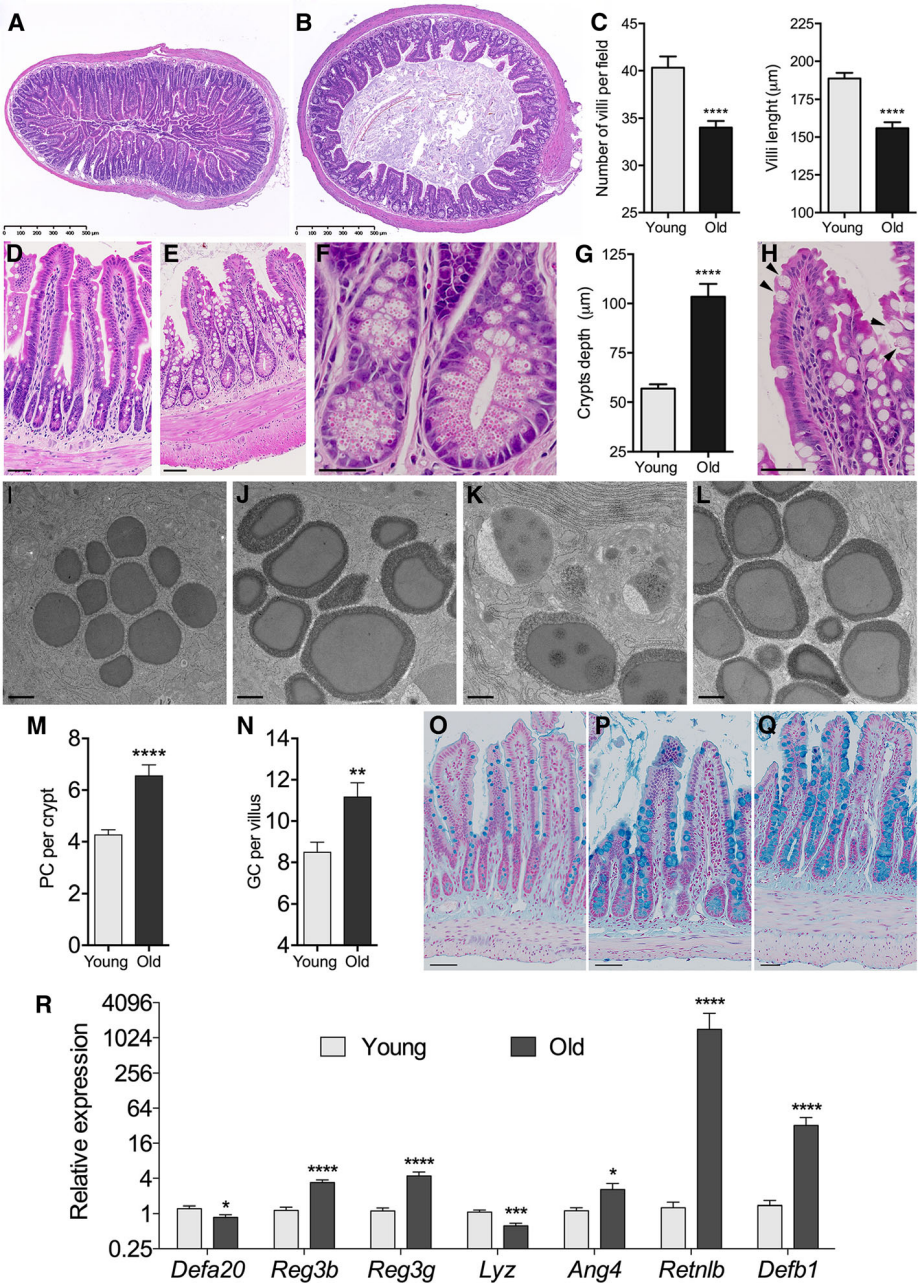
Representative H&E stained cross-sections from the ileum of young **a** and old **b** mice. **c** Average number of villi per section and villi lenght. **d**-**f** Representative H&E stained ileal sections from young **d** and old **e**-**f** mice showing villi degeneration and crypt enlargement. **g** Crypts depth in old vs. young animals. **h** Arrowheads point to goblet-like cells containing eosinophilic secretory granules. **i**-**l** Electron micrographs of Paneth cell secretory granules from a young **i** and three old animals **j**-**l**. **m** Average number of Paneth cells per crypt. **n** Average number of goblet cells per villus. **o**-**q** Alcian blue stained ileal sections from a young **o** and two old **p**-**q** animals showing goblet cell hyperplasia and intracellular accumulation of mucin. Histology pictures were taken using a NanoZoomer 2.0 slide scanner (Hamamatsu). Measurements of villi length, villi number and crypt depth were done using NDP.view 2 software (Hamamatsu). Paneth and goblet cell counts were recorded in 40–60 well-preserved villi-crypt axes per animal. **r** Relative transcript levels for ileal AMPP genes, determined by qPCR using the ddCt method corrected for primer efficiencies according to Pfaffl et al. [22], (*n* = 16 animals/group, primer sequences and methods are described in [23]). Statistical differences (Mann–Whitney U test) and are shown by asterisks (*p < 0.05, **p < 0.01, ***p < 0.001, ****p < 0.0001). Scale bars are: (**a**, **b**: 500 μm); (**d**, **e**, **o**, **p**, **q**: 50 μm); (**f**, **h**: 25 μm); (**i**, **j**, **k**, **l**: 500 nm)

Based on their recognized importance for intestinal homeostasis and defense, several AMPP from different functional classes (α-defensins, β-defensins, C-type lectins, RNAses and the cell wall-degrading enzyme lysozyme) were chosen for comparative gene expression analyses in the terminal ileum. The relative transcript levels in old animals (Fig. 1r) showed various degrees of significant differences with the younger animals (no significant differences were observed between genders). In contrast with the increased Paneth cell numbers, transcript levels for *Defa20* (a member of the α-defensins group produced exclusively by Paneth cells) and those of lysozyme (*Lyz*, another exclusive Paneth cell product) were slightly but significantly decreased. This, together with the histological and electron microscopy data is suggestive of Paneth cell dysfunction in the aged mice. In contrast, transcription of the genes *Reg3b* and *Reg3g* (coding for the C-type lectins Reg3b and Reg3g) was significantly increased, together with the resistin-like molecule beta (Relmb, gene *Retnlb*), β-defensin 1 (*Defb1*) and the RNAse angiogenin 4 (*Ang4*). The upregulation of expression of these antimicrobial genes, particularly the striking induction of β-defensin 1 and Relmb, together with the changes in Paneth and goblet cell numbers has been previously associated with gastrointestinal inflammation [14–16] and is strongly suggestive of ongoing epithelial distress in the ileum of aged mice.

The intestinal microbiota is reported to change with age, although the mechanisms underlying those changes are not fully understood (reviewed in [17]). Given the potential disrupting effect of altered AMPP expression over microbial communities, we analyzed the composition of the bacterial population by sequencing the V4 region of the 16S rRNA genes. We estimated the alpha diversity by calculating the Inverse Simpson index (an estimator of the richness in a community with uniform evenness) and the Chao1 index (Fig. 2a, b). There was a decrease in the microbial diversity of old mice, statistically significant (Kruskal-Wallis test, *p* = 0.021) with the Chao1 index, which estimates the total species richness taking into account low-abundance taxonomic groups. Linear discriminant analysis effect size (LefSE) was used to identify OTU (Operational Taxonomic Units) characterizing the two groups. The relative abundance of the most predominant OTU remained unchanged between young and old mice and analyses of molecular variance (AMOVA) of the β-diversity metrics showed no differences between the 2 groups (Table 1). However, the proportion of a group of less-abundant OTU was significantly modified (Fig. 2c). In general, several OTU belonging to the Firmicutes phylum were less abundant in old mice whereas some members of the phylum Bacteroidetes were increased (*e. g.*, the family *Porphyromonadaceae*, which has been previously associated with cognitive difficulties in elderly patients with cirrhosis [18]). The functional consequences of these changes are unclear and require further study.

**Fig. 2:**
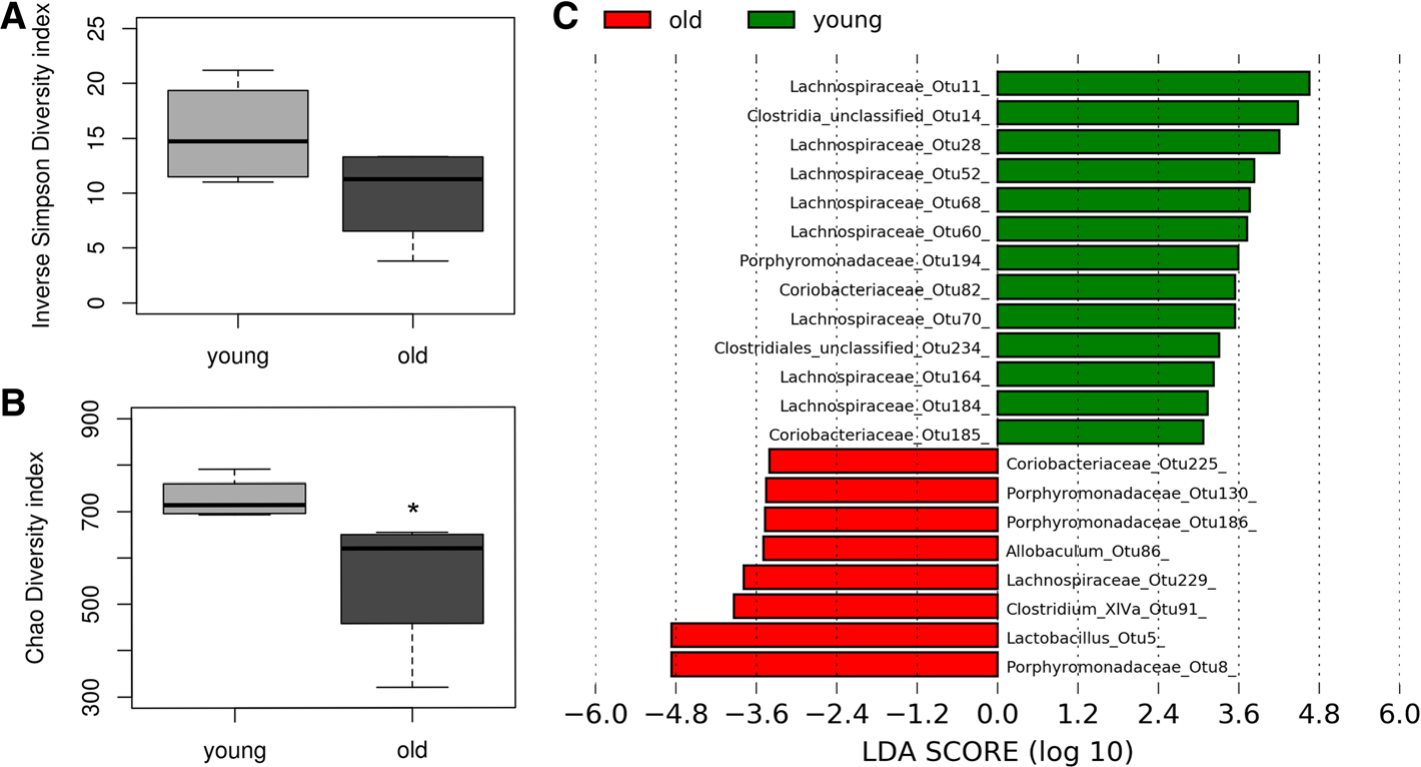
**a** Inverse Simpson index (15.41 and 9.92 in young and old mice, respectively, Kruskal-Wallis test, *p* = 0.24). **b** Chao1 index (727.85 and 553.44 in young and old mice, respectively, Kruskal-Wallis test, *p* = 0.021). **c** Linear Discriminant scores based on LefSe analysis showing the OTU more represented in young (green) and old (red) mice. Total genomic DNA extraction and sequencing of the V4 region of the bacterial 16 s rRNA gene was performed by Microbiome Insights (Vancouver, Canada). Sequences were analyzed using the software package *Mothur* (version 1.38.1) [24] according to the Standard Operating Procedure [25]. Alignment was performed using Silva database v123, reduced to the V4 region. Chimeras were removed using UCHIME [26]. Sequencing errors leading to rare variants were reduced by pre-clustering sequences into groups. Non-bacterial sequences were removed and the bacterial ones were classified using the RDB trainset no. 14. Differences in the abundance of OTUs were detected using Metastats [27]

**Table 1:** Beta diversity comparison between young and old mice shows no significant differences. Beta diversity was assessed using Unifrac metrics (weighted and unweighted) and a disimilarity matrix. Unifrac weighted incorporates phylogenetic distances and the relative abundance of the taxa, while Unifrac unweighted qualifies the membership community (presence or absence). Bray-Curtis metrics compute the dissimilarity in the communities structure. Metrics were tested for significance using a F-test (AMOVA)

Metrics	Fs value	*P* value
Unifrac weighted	2.01	0.081
Unifrac unweighted	1.19	0.153
Bray-Curtis distance	1.83	0.146

Our findings show that the homeostatic expression of AMPP is altered in the aged ileum. Those alterations were concurrent with epithelial degeneration, a slight increase in the number of Paneth and goblet cells, and mild shifts in the commensal microbial composition. However, it is currently unclear how these alterations relate to each other, namely whether they are linked or independent events and which ones might be cause or consequence. In any case, our findings open the interesting possibility of a potential contribution of altered AMPP expression to the gastrointestinal dysfunction of old age and pose the question of why and how the observed alterations are happening in the first place. Environmental factors such as diet and polymedication are thought to influence significantly the susceptibility of elderly persons to gastrointestinal disorders [1]. For example, certain diets can drive the microbiota towards a more pro-inflammatory composition and disturb its delicate equilibrium with the gut immune system effectively promoting dysfunction of the intestinal barrier [19]. However, the environmental argument does not easily hold for experimental animals kept in a controlled environment (including the diet), indicating an important involvement of fundamental host-dependent factors. Based on our data, we propose that key primary disrupting events are related to age-acquired defects in the differentiation and/or function of the secretory cell lineage, particularly Paneth and goblet cells, responsible for the secretion of multiple AMPP and mucins. Such defects would have major detrimental consequences for the integrity and function of the intestinal barrier [20, 21] and might ultimately favor the development of gastrointestinal inflammatory and physiological disorders.
